# Pathologic Prion Protein Infects Cells by Lipid-Raft Dependent Macropinocytosis

**DOI:** 10.1371/journal.pone.0003314

**Published:** 2008-10-02

**Authors:** Jehangir S. Wadia, Monica Schaller, R. Anthony Williamson, Steven F. Dowdy

**Affiliations:** 1 Department of Cellular and Molecular Medicine, University of California San Diego School of Medicine, La Jolla, California, United States of America; 2 Howard Hughes Medical Institute, University of California San Diego School of Medicine, La Jolla, California, United States of America; 3 Department of Immunology, The Scripps Research Institute, La Jolla, California, United States of America; Ordway Research Institute, United States of America

## Abstract

Transmissible spongiform encephalopathies, including variant-Creutzfeldt-Jakob disease (vCJD) in humans and bovine spongiform encephalopathies in cattle, are fatal neurodegenerative disorders characterized by protein misfolding of the host cellular prion protein (PrP^C^) to the infectious scrapie form (PrP^Sc^). However, the mechanism that exogenous PrP^Sc^ infects cells and where pathologic conversion of PrP^C^ to the PrP^Sc^ form occurs remains uncertain. Here we report that similar to the mechanism of HIV-1 TAT-mediated peptide transduction, processed mature, full length PrP contains a conserved N-terminal cationic domain that stimulates cellular uptake by lipid raft-dependent, macropinocytosis. Inhibition of macropinocytosis by three independent means prevented cellular uptake of recombinant PrP; however, it did not affect recombinant PrP cell surface association. In addition, fusion of the cationic N-terminal PrP domain to a Cre recombinase reporter protein was sufficient to promote both cellular uptake and escape from the macropinosomes into the cytoplasm. Inhibition of macropinocytosis was sufficient to prevent conversion of PrP^C^ to the pathologic PrP^Sc^ form in N2a cells exposed to strain RML PrP^Sc^ infected brain homogenates, suggesting that a critical determinant of PrP^C^ conversion occurs following macropinocytotic internalization and not through mere membrane association. Taken together, these observations provide a cellular mechanism that exogenous pathological PrP^Sc^ infects cells by lipid raft dependent, macropinocytosis.

## Introduction

The mammalian prion protein (PrP) is a GPI-anchored cell surface [1] protein expressed predominantly on neurons, neuroendocrine cells and within the lymphoreticular system [2]. PrP is considered to be the sole causative agent responsible for the transmission and development of transmissible spongiform encephalopathies such as Creutzfeldt-Jakob disease in humans, scrapie in sheep and bovine spongiform encephalopathy in cows [3]–[8]. Consequently, PrP knock-out mice, that appear phenotypically normal, remain disease-free and do not produce PrP^Sc^ following inoculation with scrapie brain homogenates [9]–[13]. The defining step in disease transmission occurs following exposure of PrP^C^ to the misfolded, infectious scrapie isoform (PrP^Sc^) resulting in the conformational conversion of wild-type host alpha-helical, protease sensitive PrP^C^ form to the beta-sheet, reduced protease sensitive PrP^Sc^ conformer [13]. However, the mechanism and cellular requirements that govern PrP^Sc^-mediated conversion of PrP^C^ remain unclear.

PrP^C^ is predominantly found on the cell surface within detergent-insoluble lipid rafts [14]. Lipid raft microdomains within the plasma membrane are composed of cholesterol and sphingolipid-rich complexes that associate with GPI-anchored proteins making them important sites for transmembrane signaling [14]–[18]. Prion proteins are continuously recycled between the cell surface and endosomal compartments, a process that requires interaction with heparan sulfate molecules and is considered to be critical to its normal and pathogenic function [19]–[23]. However, the mechanism of prion internalization remains controversial and several different endocytic pathways have been proposed [24]–[28]. Interestingly, PrP endocytosis does not appear to be a function of the GPI anchor, but instead is regulated by a phylogenetically conserved basic amino acid domain (NH_2_-KKRPKP) located within the unstructured amino terminus of the mature protein. Deletion of regions within the N-terminus of PrP^C^ or point mutations within the polybasic region have been shown to disrupt the internalization of prion protein [29]–[31]. Moreover, fusion of the NH2-terminal domain to a foreign GPI-anchored protein is sufficient to induce its endocytosis, suggesting that this property is intrinsic to this region [22].

Peptide transduction domains (PTDs), also referred to as cell penetrating peptides (CPPs), are a class of basic peptides that direct the internalization of fused macromolecules into mammalian cells [32]–[34]. The nine amino acid HIV-1 TAT and 8×Arg PTDs are prototypic PTDs that have been used to deliver a wide variety of cargoes into cells both *in vitro* and *in vivo* [32]–[34]. We previously determined that the mechanism of cationic PTD-mediated transduction into cells occurs in three phases: (i) electrostatic interaction with cell surface proteoglycans resulting in (ii) stimulated uptake by lipid-raft dependent macropinocytosis and (iii) escape from the endosomal compartment into the cytoplasm [35], [36]. In scanning the signal peptide cleaved PrP^C^ (residues 23–231) amino acid sequence, we identified putative transduction domains in the amino terminus between residues 23–29 and 100–109. Similar to the TAT PTD, we found that uptake of exogenous recombinant PrP^C^ protein in N2a neuroblastoma cells was sensitive to cholesterol depleting agents and macropinocytosis inhibitors. We also found that inhibition of macropinocytosis was sufficient to prevent conversion of PrP^C^ to PrP^Sc^ indicating that pathogenic amplification of PrP^Sc^ occurs following cellular internalization by macropinocytosis. Thus, the internalization of PrP and the TAT PTD appear to share a conserved macropinocytotic mechanism.

## Results

**Figure 1:**
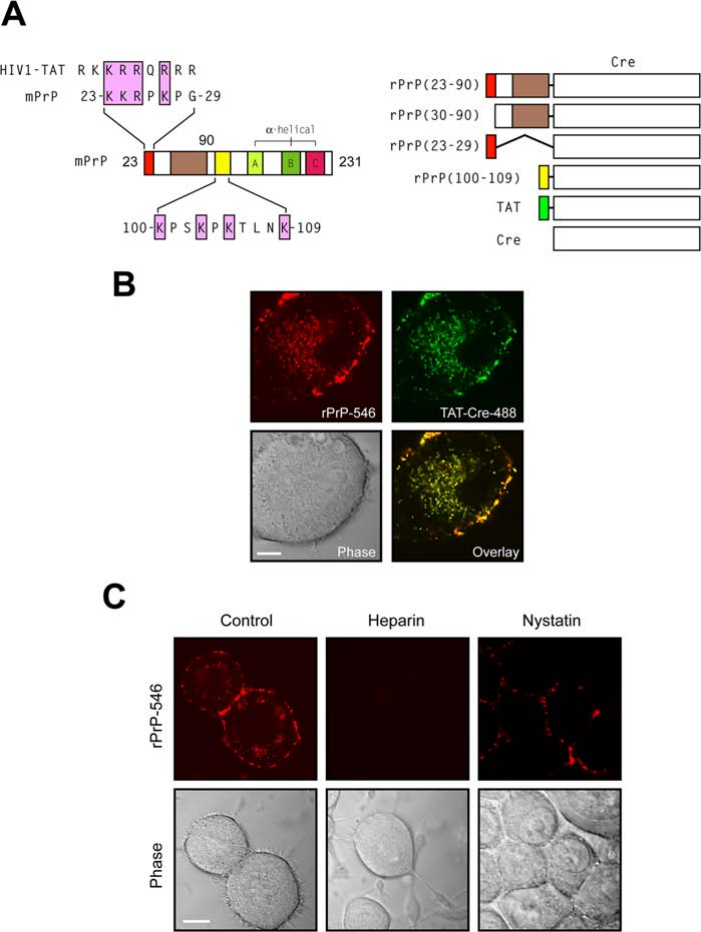
Exogenous rPrP is endocytosed into cells and co-localizes with TAT-Cre. (A) Alignment of putative PrP^C^ transduction domain with the HIV-1 TAT PTD and schematic of rPrP-Cre recombinase fusion proteins. (B) Co-localization of rPrP (residues 23–231) and TAT-Cre. N2a cells were treated with rPrP-Alexa546 (red) and TAT-Cre-Alexa488 (green) for 1 hr, then assayed by live cell confocal microscopy. Yellow fluorescence indicates areas of co-localization. Scale bar = 5 µm. (C) N2a cells pre-incubated for 30 min with 50 µg/mL heparin or 5 mM nystatin followed by rPrP-Alexa546 protein incubation for 2 hr prevented surface binding and internalization, respectively. Scale bar = 10 µm.

To compare the endocytic uptake of exogenous PrP with the TAT PTD, we co-incubated murine N2a neuroblastoma cells with fluorescently-labeled recombinant murine PrP (rPrP)-546 (red) and TAT-Cre recombinase-488 (green) reporter proteins (Figure 1A, B). Confocal microscopy on live cells showed that both rPrP-546 and TAT-Cre-546 proteins rapidly accumulated within the cell and co-localized within the same intracellular vesicles (Figure 1B), suggesting that exogenous rPrP protein and TAT PTD are internalized within the same intracellular compartments in these cells. The initial step in TAT PTD transduction requires an ionic interaction with cell surface glycosaminoglycans (GAGs) that is thought to be required for stimulation of uptake [32]–[34]. Likewise, prion proteins have previously been shown to bind to GAGs, including chondroitin sulfate, hyaluronic acid and heparin, within lipid raft microdomains on the plasma membrane [19], [20], [37] and deletion of PrP^C^ residues within the NH2-terminal have been shown to abrogate these interactions [38]. Similarly, we found that incubation of N2a cells with anionic heparin prevented membrane binding and endocytosis of exogenous, fluorescently labeled rPrP (Figure 1C). Transmission of disease is thought to involve PrP^C^ located within lipid rafts since cholesterol depletion at the cell surface has been reported to attenuate the conversion of PrP^C^ to the scrapie form [19], [39]. We found that disruption of lipid raft microdomains by nystatin sequestration of cholesterol both reduced rPrP membrane binding and blocked internalization, suggesting that extracellular rPrP was also directed to lipid raft microdomains and that localization to rafts was required for uptake of extracellular rPrP (Figure 1C). Remarkably, these observations paralleled closely the macropinocytotic uptake mechanism of arginine-rich peptide transduction domains [35], [36].

**Figure 2:**
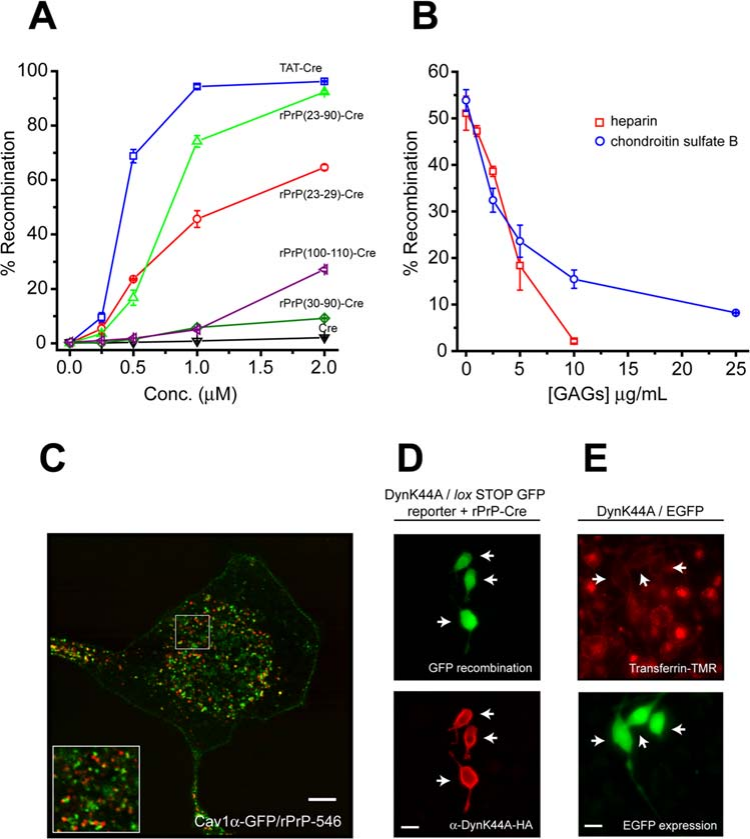
Cellular uptake of rPrP occurs by endocytosis. (A) Reporter cells containing *lox*P-STOP-*lox*P GFP reporter gene were treated with indicated proteins for 1 hr, incubated overnight, then assayed for GFP expression by flow cytometry (±SD). (B) Reporter cells were treated with rPrP (23–90)-Cre in the presence of heparin or chondroitin sulfate B, washed, incubated overnight, then assayed for GFP-positive cells by flow cytometry (±SD). (C) N2a cells were transfected with Cav1α-GFP expression plasmid, followed 48 hr later by treatment with rPrP-546 (red) and live cell confocal microscopy. Scale bar = 5 µm. (D) N2a cells co-transfected with pDyn^K44A^-HA dominant-negative and pZ/EG *lox*P-stop-*lox*P GFP reporter plasmids were treated with rPrP (23–90)-Cre protein. Scale bar = 25 µm. (E) N2a cells co-transfected with pDyn^K44A^-HA and pEGFP (constitutive GFP expression) plasmids (10∶1) were incubated with fluorescent transferrin-TMR (red) as a positive control for Dyn^K44A^ activity. Arrows indicate Dyn^K44A^/EGFP transfected cells. Scale bar = 20 µm.

Sequence analysis of the NH2-terminus of PrP revealed the presence of two putative PTDs with homology to the polybasic TAT transduction domain (Figure 1A). To ascertain the amino acid sequence requirements within the amino terminus for PrP entrance into cells, we generated recombinant fusion proteins with different PrP domains and Cre DNA recombinase as a reporter for cellular internalization and endosomal escape (Figure 1A). Previously we have shown that extracellular recombinant TAT-Cre fusion protein is rapidly internalized into cells and translocates to the nucleus where it can excise a transcriptional termination DNA segment from a *lox*P-stop-*lox*P GFP reporter gene to allow GFP expression [35]. Consistent with TAT-Cre addition, treatment of reporter cells with rPrP (23–90)-Cre, or the putative N-terminal PrP transduction domain alone, rPrP (23–29)-Cre, resulted in the concentration-dependent recombination and expression of GFP (Figure 2A). Surprisingly given the limited basic composition, rPrP (23–90)-Cre entered cells at a similar level as the TAT PTD. However, rPrP (100–110)-Cre fusion showed a significantly weaker ability to enter cells, achieving a recombination maximum of only 27% at 2 µM protein (Figure 2A). In contrast, both rPrP (30–90)-Cre and control Cre protein treatment failed to induce recombination above background levels (Figure 2A). Co-incubation of cells with rPrP (23–90)-Cre protein and soluble GAGs heparin or chondroitin sulfate B, inhibited protein uptake into cells and subsequent DNA recombination in a dose-dependent manner (Figure 2B). These observations confirm the presence of a strong transduction domain at the N-terminus of PrP (residues 23–29) and a weaker domain between residues 100–110 that are sufficient for not only endocytic uptake, but also subsequent cytoplasmic escape of extracellular rPrP. Interestingly, the entire N-terminal domain of PrP (23–90) was more effective than just the basic PrP (23–29) domain at inducing recombination, suggesting the presence of additional elements that may enhance transduction and/or escape into the cytoplasm.

Endocytic uptake of endogenous PrP^C^ is controversial and has previously been reported to occur through either clathrin [24] or caveolar-dependent forms of endocytosis [20], [37]. However, cellular uptake of the TAT PTD does not occur through either mechanism [35], [40]. To examine the endocytic mechanisms of extracellular rPrP uptake, we treated N2a cells expressing GFP tagged caveolin-1-α, a marker of caveolae, with fluorescent rPrP-546. Parallel to TAT PTD uptake, the vast majority of rPrP-546 (red) containing vesicles did not co-localization with caveolae by confocal imaging of live cells (Figure 2C). Of the limited number of co-localized (yellow) vesicles, the majority are not completely circular (spherical), but partial circles indicating that two independent vesicles above/below each other are overlapping and thereby appear to be co-localized. Multiple forms of endocytosis, including both clathrin and caveolar mediated endocytosis, require dynamin GTPase activity for vesicle formation at the cell surface. Expression of a dominant-negative dynamin-1 (Dyn^K44A^) has been used to effectively and selectively block these endocytic pathways [42]. Consistent with the absence of Caveolin-1 co-localization, expression of Dyn^K44A^ in N2a cells containing a *loxP*-stop-*loxP* GFP reporter plasmid, failed to block cellular uptake of rPrP (23–90)-Cre reporter protein, recombination and GFP expression (Figure 2D). As a positive control, Dyn^K44A^ expression inhibited uptake of a clathrin-mediated endocytosis marker, fluorescent-labeled transferrin (Figure 2E). These observations demonstrate that internalization of extracellular rPrP occurs through a lipid raft-dependent process that is independent of both caveolar- and clathrin-mediated endocytosis.

**Figure 3:**
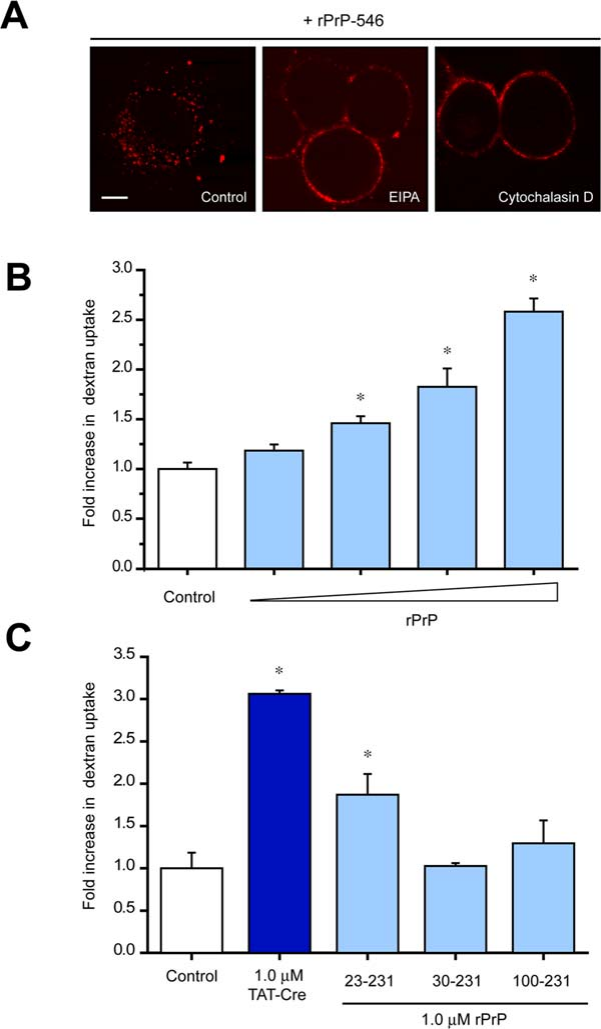
Cellular uptake of rPrP occurs by macropinocytosis. (A) N2a cells were pretreated with either 100 µM EIPA or 5 mM cytochalasin D for 30 min prior the addition of 2.0 µM fluorescently labeled recombinant rPrP-Alexa546. After 2 hr, cells were washed and analyzed by live cell confocal microscopy. Scale bar = 10 µm. (B) N2a cells were treated with 0.5 mg/mL 70 kDa neutral dextran-FITC for 30 min in the presence of increasing concentrations of rPrP (0, 0.25, 0.5, 1.0 or 2.0 µM). Fluid phase uptake of dextran was measured by flow cytometry (values±SD, Student's T-test: ** = p<0.02, *** = p<0.002). (C) N2a cells were treated with 0.5 mg/mL 70 kDa neutral dextran-FITC for 30 min in the presence of 1.0 µM TAT-Cre, rPrP (23–231), rPrP (30–321) or rPrP (100–231). Fluid phase uptake of dextran was measured by flow cytometry.

Previously we determined that TAT-fusion proteins and peptides enter cells by lipid raft-dependent macropinocytosis [35], [41], a specialized actin-dependent, fluid phase endocytic process [41]. To examine the potential involvement of macropinocytosis in rPrP uptake, we treated N2a cells with macropinocytosis inhibitors EIPA, an analogue of amiloride that inhibits a Na^+^/H^+^ exchange specific for macropinocytosis [42], or cytochalasin D, an F-actin elongation inhibitor [43], prior to incubation with fluorescent-labeled rPrP-546 and measured internalization using live cell confocal imaging. Treatment of N2a cells with either macropinocytosis inhibitor prevented the cellular uptake of rPrP-546, but did not block its cell surface association (Figure 3A). Cell surface binding of TAT-fusion proteins has been shown to stimulate macropinocytotic uptake [35], [36], [40]. To determine whether extracellular rPrP cell surface binding can similarly induce macropinocytosis, we co-incubated N2a cells with a fluorescent marker of fluid phase macropinocytosis, 70-kDa dextran-FITC [44], and increasing concentrations of rPrP (Figure 3B). Treatment of cells with rPrP induced a significant (p<0.02−0.002), concentration-dependent increase in fluorescent dextran, fluid-phase uptake over steady-state control levels (Figure 3B). Conversely, both rPrP (residues 30–231) minus the N-terminal PTD or rPrP (residues 100–231) minus the weak PTD failed to stimulate macropinocytotic uptake of dextran (Figure 3C). Taken together, these observations exclude both clathrin and caveolar endocytosis as the mechanism of exogenous rPrP uptake, and demonstrate that rPrP can stimulate it's own uptake by lipid raft-mediated macropinocytosis.

**Figure 4:**
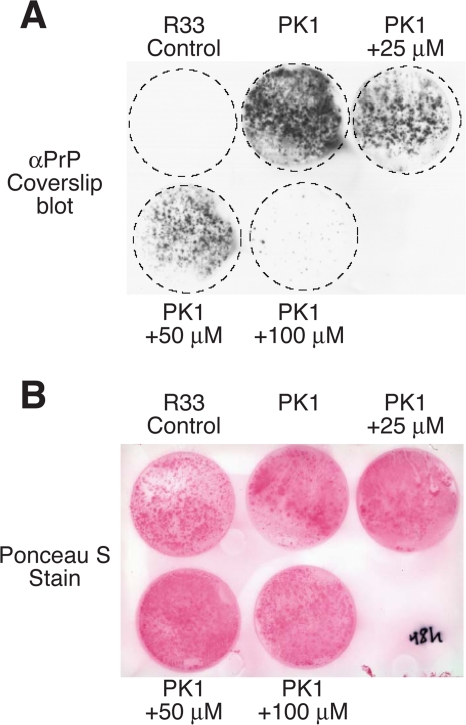
Pathological PrP^Sc^ conversion of PrP^C^ requires macropinocytosis. (A) N2aPK1 cells were exposed to 10^−5^ dilution of strain RML scrapie PrP^Sc^-infected murine brain homogenate and increasing concentrations of the macropinocytosis inhibitor EIPA (25, 50 and 100 µM) for 48 hr. Infected cells were then grown on cover slips, blotted, digested with proteinase K (PK) and probed with anti-PrP antibodies (A) and Ponceau S staining (B).

PrP^C^ and pathological PrP^Sc^ have a markedly different COOH-terminal structural conformation with PrP^Sc^ having extensive β-sheet content [3]–[8]. To determine if pathological PrP^Sc^ infects cells by macropinocytosis and if internalization is necessary for conversion of endogenous PrP^C^ to the PrP^Sc^ form, we treated scrapie susceptible N2aPK1 [12], [45] cells with a 10^−5^ dilution of strain RML PrP^Sc^-infected murine brain homogenates and the macropinocytosis inhibitor EIPA. Consistent with previous reports [12], [45], treatment of N2aPK1 cells with strain RML PrP^Sc^-infected brain homogenates resulted in conversion of PrP^C^ into the proteinase K resistant PrP^Sc^ form, whereas control non-susceptible N2aR33 cells were resistant to pathogenic conversion (Figure 4). Surprisingly, co-incubation of N2aPK1 cells with strain RML PrP^Sc^-infected brain homogenate in the presence of EIPA resulted in an EIPA concentration-dependent inhibition of PrP^Sc^ conversion of PrP^C^ to the pathologic form (Figure 4). Importantly, the concentrations of EIPA used did not inhibit the cellular density of N2aPK1 cells (Figure 4B). The requirement of macropinocytosis for pathogenic conversion of PrP^C^ suggests that macropinocytotic uptake of PrP^Sc^ into cells is a critical step in the propagation of disease.

## Discussion

Fatal transmissible spongiform encephalopathies are fatal neurodegenerative diseases that occur following host exposure of PrP^Sc^ contaminated tissue resulting in conversion of PrP^C^ form into the disease causing, partially protease resistant PrP^Sc^ form [3]–[8]. Fundamental mechanistic questions of how exogenous PrP^Sc^ protein infects cells, is transported between cells and where conversion of PrP^C^ to the pathological PrP^Sc^ form takes place remain unclear. However, the observations presented here suggest that PrP^Sc^ and the HIV TAT PTD share similar characteristics, including cell surface association, cellular uptake, intracellular localization and that these similarities may account for properties of PrP^Sc^ during disease propagation. Consistent with cellular uptake of a short N-terminal leader PrP peptide [46], we found that mature (processed), full length PrP^C^ contains a strong N-terminal transduction domain between residues 23–29 and a much weaker transduction domain at residues 100–110. Similar to the TAT PTD, these cationic PrP domains are sufficient to direct the uptake, internalization and cytoplasmic release of a PrP-CRE recombinase fusion protein, providing a molecular basis for PrP^Sc^ infection.

The endocytic pathway involved in PrP^Sc^ uptake is unclear. The observations presented here using dominant-negative dynamin K44A and inhibitors of macropinocytosis indicate that, similar to the TAT protein transduction domain, endocytic uptake of PrP occurred by lipid-raft dependent macropinocytosis, and not caveloin or clathrin mediated endocytosis. Consistent with PTD uptake, extracellular PrP binding to the cell surface could be competed with soluble GAGs. Interestingly, interaction of full length PrP with the cell surface was sufficient to stimulate macropinocytosis, while deletion of the N-terminal transduction domain abrogated this effect. Although the GPI anchor within PrP is a determinant of lipid-raft association, PrP has been shown to remain raft associated when the GPI anchor was deleted and was only abolished by deletion of the N-terminal 23–90 amino acids further supporting the notion that this domain is important in cellular raft targeting of exogenous PrP [47]. Interestingly, a PrP variant, PrP27–30, that lacks the N-terminal transduction domain, but retains the weaker PTD at residues 100–110, retains a low level ability to cause pathogenic conversion of PrP^C^, suggesting that the second lysine-rich transduction motif may be sufficient in the absence of the N-terminal domain to direct cellular entry. Small amounts of cytoplasmic PrP have been reported to cause neurotoxicity [5]–[7], [48]. Consistent with this notion, our experiments using PrP-Cre reporter fusion proteins as a phenotypic marker of endosomal escape suggest that, at least, a small proportion of extracellular PrP that is internalized can escape from macropinosomes into the cytoplasm.

During the normal course of infection, host PrP^Sc^ exposure is thought to occur following oral uptake of contaminated material. Although PrP^Sc^ entry into the nervous system and subsequent neuronal infection and propagation are critical steps in the pathogenesis of disease, the mechanistic basis remains poorly understood. Oral administration of a TAT-beta-galactosidase reporter protein in mice is sufficient for distribution and enzyme activity within most tissues, including a small amount detected in the brain [49]. The presence of a functional PTD in prion protein provides a mechanistic basis underlying infectious PrP^Sc^ transport between cells. Indeed, broad based polyanionic compounds, such as heparin and PEI, have been reported to inhibit PrP^Sc^ infection of cells [50]–[52], potentially by sequestration of the N-terminal PTD. However, specific inhibitors of macropinocytosis may potentially function more efficaciously to prevent initial cellular uptake of PrP^Sc^. Interestingly, Lofgren et al [53] found that a PrP peptide corresponding to the unprocessed N-terminal signal sequence and transduction domain (residues 1–28) specifically blocked PrP^Sc^-mediated conversion of PrP^C^, whereas other PTD peptides did not block conversion, suggesting a specific downstream effect of cellular uptake.

In conclusion, the observations presented here suggest that prion protein contains transduction motifs similar to those found within HIV-1 TAT and that these domains provide a molecular mechanism for pathological PrP^Sc^ infection of cells by macropinocytosis and subsequent conversion of PrP^C^.

## Materials and Methods

### Recombinant Proteins

rPrP cloned in a pET28 vector (Novagen) was expressed in BL21 pLysS cells (Novagen) after induction with IPTG for 3 hr. Cells were resuspended in cold buffer W (50 mM Tris pH 8.0, 250 mM NaCl, 5 mM EDTA, 1 mM PMSF, 10 µg/mL leupeptin, 0.1 mM aprotinin,10 µg/mL DNase 1, 10 µg/mL lysozyme), sonicated and inclusion bodies collected by centrifugation at 30,000×g, 20 min and solubilized in buffer G (6 M GdmCl, 20 mM Tris pH 8.0, 50 mM Na_2_HPO_4_, 100 mM NaCl, 10 mM reduced glutathione, 10 mM imidazole). Cleared lysates were incubated overnight at RT with shaking, purified on Ni-NTA column, washed with a gradient of buffer G and buffer B (10 mM Tris pH 8.0, 100 mM Na_2_HPO_4_, 0.1 mM oxidized glutathione, 10 mM imidazole) at ratios of 6∶0, 5∶1, 4∶2, 3∶3, 4∶2, 5∶1 and 0∶6, respectively. rPrP was eluted in 20 mM Tris pH 8.0, 1 M imidazole and an on-column oxidation was repeated twice. Fractions were buffer exchanged into 50 mM Hepes pH 7.0, 100 mM NaCl and 5% glycerol and concentrated by ultrafiltration. rPrP (23–90)-Cre, rPrP (30–90)-Cre, rPrP (23–29)-Cre, rPrP (100–109)-Cre, TAT-Cre, and control Cre were purified as previously described [35]. rPrP and TAT-Cre proteins were conjugated with Alexa546 or Alexa488 (Molecular Probes) so that the degree of labeling was approximately 1.

### Recombination Assays

Reporter T cells^10^ containing an integrated *loxP*-STOP-*loxP* GFP expression gene were treated with recombinant protein in the presence/absence of heparin (Sigma) or chondroitin sulfate B (Sigma) RPMI for 1 hr at 37°C, 5% CO_2_. Cells were trypsinized, washed 2× in PBS and replated in RPMI+10% FBS for 18 h followed by FACS for GFP positive cells. Cell death was measured by propidium iodide staining, and using flow cytometry forward/side analysis.

### Confocal microscopy

Murine N2a neuroblastoma cells were grown on glass coverslips and exposed to 2.0 µM rPrP-Alexa546 for 2 hr, washed and live cell images were acquired at a depth through the middle of the nucleus using a BioRad MRC1024 confocal microscope. To determine colocalization with caveolae, N2a cells were transiently transfected with 0.2 µg caveolin-1α-GFP expression vector using Fugene-6, washed and incubated with rPrP-Alexa546 for 2 hr. N2a cells were pretreated with either 50 µg/mL heparin (Sigma), 5 mM nystatin (Sigma), 100 µM cytochalasin D (Sigma) or 100 µM EIPA (Sigma) for 30 min, prior to adding 2.0 µM rPrP-Alexa546 for 2 hr. For co-localization studies, N2a cells were treated with 2.0 µM rPrP-Alexa546 and 2.0 µM TAT-Cre-Alexa488. After 2 hr, cells were washed and corresponding fluorescent confocal images for rPrP-Alexa546 fluorescence (PMT1) and TAT-Cre-Alexa488 fluorescence (PMT2) were obtained.

### Dynamin-1 (K44A)

N2a cells were transfected at a ratio of 10∶1 with Dynamin^K44A^-HA (pDyn^K44A^-HA) expression plasmid (S. Schmid, Scripps Research Institute) and pZ/EG *loxP*-STOP-*loxP* GFP expression vector (A. Nagy, Univ. Toronto). After 24 hr, cells were treated with 2.0 µM rPrP (23–90)-Cre for 1 h, trypsinized, washed, replated in DMEM+10% FBS for 18 h and analyzed for GFP by FACS. Immunohistochemistry using anti-HA antibody (Babco) followed by anti-mouse TRITC secondary antibody (Jackson Labs) was used to verify Dyn^K44A^ expression. Control Dyn^K44A^ and pEGFP vector (Stratagene) (10∶1) expressing cells were incubated in serum-free media for 4 hr prior to addition of 25 µg/mL transferrin conjugated tetramethylrhodamine (Molecular Probes) for 15 min.

### Quantification of Macropinocytosis

N2a cells were incubated in DMEM+(serum-free DMEM, 0.1% BSA and 10 mM Hepes pH 7.4) at 4°C for 30 min. To measure macropinocytosis, 0.5 mg/mL 70 kDa neutral dextran-FITC (Molecular Probes) was added to cells treated with increasing concentrations of recombinant rPrP (0, 0.25, 0.5 1.0, or 2.0 µM), incubated for 30 min and analyzed by FACS. Background dextran fluorescence uptake was inhibited by incubation at 4°C for 30 min. Fold increase in dextran uptake was calculated after subtracting background fluorescence from each sample.

### Cell-based PrP^Sc^ infectivity assay

Except were noted, infectivity assay was performed as described [12], [45]. Briefly, 1×10^4^ susceptible N2aPK1 cells (gift from C. Weissmann) and resistant N2aR33 cells, maintained in opti-MEM (Gibco) plus 10% FBS, were exposed to a 10^−5^ dilution of strain RML PrP^Sc^-infected murine brain homogenates for 48 hr in the presence or absence of 25, 50, 100 µM EIPA (Sigma). Cells were grown to confluence, washed and split 1∶10. Replating at 1∶10 was repeated twice. After the last passage 5×10^4^ cells were plated onto 25 mm Thermanox coverslips (Nunc, Fischer Scientific), grown for 4 days and then blotted onto a nitrocellulose membrane (Biorad, 0.45 µm pore size) soaked in lysis buffer (1% Triton ×100, 0.5% deoxycholate, 150 mM NaCl, 50 mM Tris-HCl, pH 8.0) as described [12], [46]. The membranes were dried for 1 h at 37°C, incubated with 0.5 µg/mL PK in lysis buffer for 90 min at 37°C followed by treatment with 3 M guanidinium thiocyanate, 10 mM Tris-HCl (pH 8.0) for 10 min. After washing, the membranes were blocked in 5% non-fat dry milk and probed with 6H4 anti-PrP antibody (1∶1000, Prionics, Zurich) followed by HRP-conjugated anti-mouse IgG1 antibody. PrP^Sc^ positive colonies were visualized using Supersignal West Pico ECL reagent (Pierce Biotechnology).
